# The helix-loop-helix transcriptional regulator Id4 is required for terminal differentiation of luminal epithelial cells in the prostate

**DOI:** 10.18632/oncoscience.524

**Published:** 2021-03-24

**Authors:** Dhanushka Hewa Bostanthirige, Shravan K. Komaragiri, Jugal B. Joshi, Majid Alzahrani, Isha Saini, Sanjay Jain, Nathan J. Bowen, Matthew C. Havrda, Jaideep Chaudhary

**Affiliations:** ^1^Center for Cancer Research and Therapeutics Development, Clark Atlanta University, Atlanta GA, USA; ^2^Lifeline Pathology Lab and Diagnostic Center, Karnal, India; ^3^Morehouse School of Medicine, Atlanta, GA, USA; ^4^Geisel School of Medicine, Hanover, NH, USA

**Keywords:** ID4, Pten, androgen receptor, prostate cancer

## Abstract

Inhibitor of differentiation 4 (Id4), a member of the helix-loop-helix family of transcriptional regulators has emerged as a tumor suppressor in prostate cancer. In this study we investigated the effect of loss of Id4 (*Id4-/-*) on mouse prostate development. Histological analysis was performed on prostates from 25 days, 3 months and 6 months old *Id4-/-* mice. Expression of Amacr, Ck8, Ck18, Fkbp51, Fkbp52, androgen receptor, Pten, sca-1 and Nkx3.1 was investigated by immunohistochemistry. Results were compared to the prostates from *Nkx3.1-/-* mice. *Id4-/-* mice had smaller prostates with fewer and smaller tubules. Subtle PIN like lesions were observed at 6mo. Decreased Nkx3.1 and Pten and increased stem cell marker sca-1, PIN marker Amacr and basal cell marker p63 was observed at all ages. Persistent Ck8 and Ck18 expression suggested that loss of Id4 results in epithelial commitment but not terminal differentiation in spite of active Ar. Loss of Id4 attenuates normal prostate development and promotes hyperplasia/ dysplasia with PIN like lesions. The results suggest that loss of Id4 maintains stem cell phenotype of “luminal committed basal cells”, identifying a unique prostate developmental pathway regulated by Id4.

## INTRODUCTION

Id4, a member of the helix loop helix family of transcriptional regulators (that also includes the paralogues Id1, Id2 and Id3), has emerged as a key determinant for prostate development and differentiation. Genetic ablation of *Id4* attenuates prostate development and branching morphogenesis and impacts normal development of other sex accessory glands such as seminal vesicles [1]. In addition to its role in prostate development, Id4 also regulates mammary gland branching morphogenesis [2], glial differentiation [3] and neuronal development [4].

Epigenetic silencing of ID4 is frequently observed in prostate and many other cancers (reviewed in [5]). Decreased expression of ID4 is also associated with development of castration resistant prostate cancer (CRPC) and overall survival [6]. Thus, the role of ID4 in prostate development and as a prostate cancer tumor suppressor is well established. Despite these recent advances, the underlying molecular mechanism of action of ID4 remains elusive. Recent studies have suggested that ID4 may act as a chaperone or a co-chaperone that promote the assembly of large transcriptional complexes for example FKBP52-AR [7] and/or regulate acetylation of p53 [8].

Id4 is expressed primarily in the luminal epithelial cells in normal adult mice and human prostates [1, 9]. Genetic ablation of *Id4* results in smaller prostate and fewer tubules [1]. Although, regions of prostatic hyperplasia were clearly observed that suggested early prostatic intraepithelial neoplasia (PIN) lesions, full blown cancerous regions were not observed in *Id4-/-* mice prostate. The associated proliferation markers such as Id1, Ki67 and Myc were clearly upregulated in *Id4-/-* prostates as compared to wild type counterparts. The expression of tumor suppressor Pten was below detection and correspondingly the level of pAkt was significantly higher in *Id4-/-* mice prostates. The prostate development associated homeobox transcription factor and a tumor suppressor Nkx3.1 was also not expressed or was below detection in *Id4-/-* mice prostates. Collective results suggested that *Id4-/-* knockout (KO) mice display complex phenotypes that resemble or mimic Pten and Nkx3.1 combined KO in many respects but with no clear cancerous phenotype or prostatic hyperplasia observed in Nkx3.1+/-;Pten+/- [10] or Nkx3.1-/- [11] mice respectively. These results led us to hypothesize that Id4 may regulate key prostate developmental pathways, independent of Pten and Nkx3.1. A developmental block due to loss of Id4 may represent a threshold which need to be overcome for the development of prostate cancer even in the absence of major tumor suppressor Nkx3.1 and Pten and over-expression of myc. To further explore the developmental pathways regulated by Id4 and building on our earlier studies performed only on 6weeks old *Id4-/-* mice prostate, in this study we investigated the prostate development from 25 days old (d), 3 months (mo) and 6 mo old prostates. The results presented herein demonstrates that loss of Id4 results in epithelial cells with a complex phenotype that express stem cell (Sca-1), secretory epithelial (probasin/Pbsn), Ck8) and early PIN lesion (Amacr) markers. Starting at 3mo, epithelial stratification and tufting was clearly visible in the dorsal prostate lobes. Our results suggest that loss of Id4 may result in a differentiation arrest of epithelial cells that maintain stem cell and differentiation markers resulting in low penetrance PIN by 3 mo.

## Results

### Loss of Id4 attenuates prostate development

**Figure 1:**
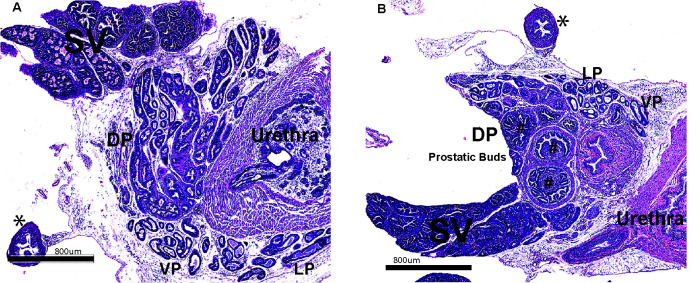
Prostate histology (H&E) from 25day old wild type (A) and Id4 knockout (*Id4-/-*) mice. Various identifiable regions of the prostate are marked: SV: Seminal Vesicle, DP: Dorsal prostate, VP: Ventral Prostate, LP: Lateral prostate, *: Vas Deferens, #: Prostatic Buds. The magnification scale is indicated.

**Figure 2:**
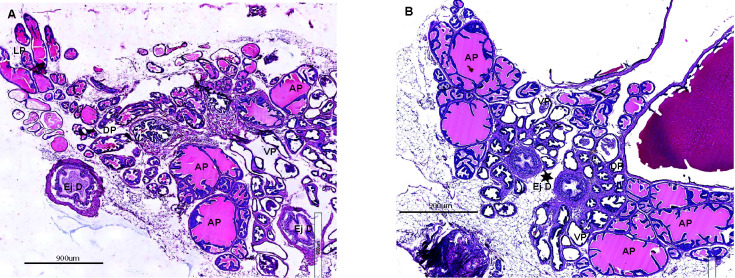
Prostate histology (H&E) from 3month old wild type (A) and Id4 knockout (*Id4-/-*) mice. Various identifiable regions of the prostate are marked: DP: Dorsal prostate, VP: Ventral Prostate, LP: Lateral prostate, AP: Anterior Prostate, Ej D: Ejaculatory duct. The magnification scale is indicated.

**Figure 3:**
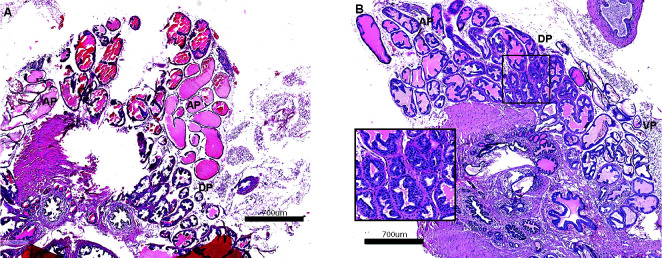
Prostate histology (H&E) from 6month old wild type (A) and Id4 knockout (*Id4-/-*) mice. Various identifiable regions of the prostate are marked: DP: Dorsal prostate, VP: Ventral Prostate, AP: Anterior Prostate. The inset is the section of DP that clearly demonstrates epithelial stratification with a tufting pattern indicative of early PIN lesions. The magnification scale is indicated.

As expected, the prostate gland and genital tract in 25d old (see Seminal Vesicles, SV, in Fig. 1) and adult *Id4-/-* mice (3month, Fig. 2 and 6 month Fig. 3) continued to be severely impaired that was consistent with our observations on 6 weeks old mice [1]. All lobes in *Id4-/-* mice displayed attenuated development both in number of tubules and size. Lobe specific developmental defects were also observed in *Id4-/-* mice, specifically in the dorsal prostate as discussed below.

The prostate from 25d old *Id4-/-* mice provided significant insight into the role of Id4 in early prostate development. Instead of multiple tubules as observed in the wild type mice (Fig. 1A), the dorsal prostate (DP) in *Id4-/-* mice at 25d only showed 2-3 large prostatic bud like structures surrounded by a densely cellular mesenchymal layer (Fig. 1B, shown by #). The mesenchymal regions were also observed inside the buds suggesting an area of active transition to form tubules. Thus, DP was represented by poor differentiation of prostatic ducts and glandular acini. In the wild type DP, ductal branching was well advanced including thinning of the mesenchyme around the ducts. During rat prostate development, anatomically distinct lobes are visible by day 5 after birth. Varying degrees of ductal branching morphogenesis which are distinctly different in different lobes also appear by day 5. At day 10, ductal branching is well-advanced in all prostatic lobes that is associated with the thinning of the surrounding mesenchyme [12]. These observations led us to conclude that at the 25d chronological age, the *Id4-/-* mouse prostate is closer to day 5 developmental age.

At 3mo, the smaller tubule size in the *Id4-/-* mice was consistent with small prostate size. The branching morphogenesis appeared complete at 3mo since no prostatic buds as seen in 25d *Id4-/-* mice were present (Fig. 2). Evidence of any PIN lesions were also not apparent even though epithelial stratification was observed in multiple tubules.

Hyperplastic regions within the tubules of 6mo old *Id4-/-* dorsal prostate were observed (Inset, Fig. 3B). The nuclei of *Id4-/-* mice in these regions appeared hyperchromatic as compared to the homogenous chromatin found in wildtype nuclei further suggesting hyperplasia and dysplasia (Fig. 3B inset). These regions are reminiscent of early PIN, which is considered as a precursor of invasive prostate carcinoma in humans and genetically engineered mouse models of prostate cancer [13, 14]. These results suggested that loss of *Id4* only leads to localized hyperplasia/ early PIN lesions but not prostate cancer even at 6mo of age.

### Id4 expression in the normal prostate

**Figure 4:**
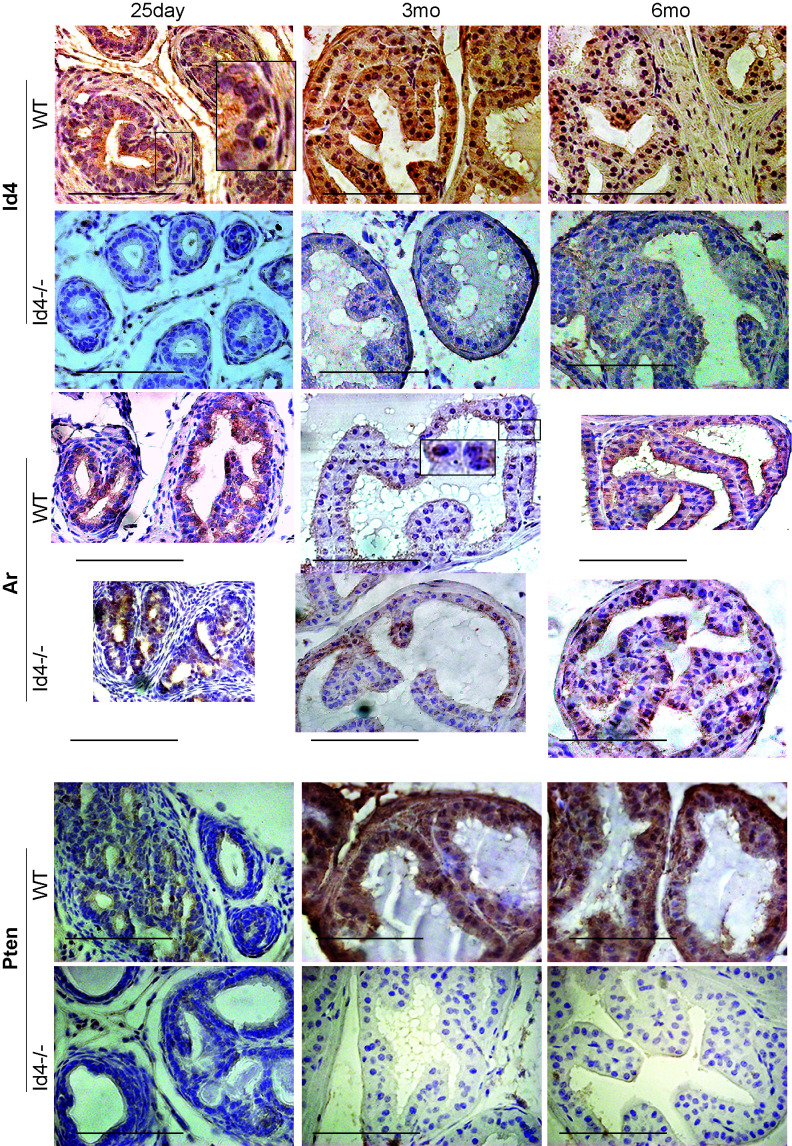
Immuno-histochemical localization of Id4 (top panel), androgen receptor (Ar, middle panel), and Pten (bottom panel) in *Id4-/-* (Id4 knockout) and wt (wild type) prostates from 25d, 3m and 6m old mice. The positive immune-reactivity is identifiable as brown staining. The bar in each panel is 100um. The insets represent an enlarged boxed region. The blue staining represents nuclei stained with hematoxylin. Representative data from multiple fields and 3 different experiments is shown.

Id4 was expressed as early as 25 days in the developing prostate (Fig. 4). Id4 expression appears to be limited to the tubules. (Fig. 4). These results suggest that Id4 is highly expressed in the adult mouse prostate glandular epithelial cells whereas mesenchymal cell also express Id4 in the developing 25d old prostates.

### Androgen Receptor (Ar) is predominantly nuclear in *Id4-/-* mice

The Ar expression appeared more robust and localized in the *Id4-/-* mice as compared to wt (Fig. 4). At 25d, the nuclear Ar expression was observed in a few cells across the tubules in *Id4-/-* and appeared more diffused in wt. This expression pattern remained consistent, that is, more diffuse than nuclear in wt at all time points (Fig. 4). In contrast, the Ar expression appeared more nuclear localized in *Id4-/-* mice prostates represented by intense nuclear Ar staining (Fig. 4).

### Loss of Pten expression in *Id4-/-*

We had reported earlier that Pten expression is essentially absent or below detection in 6wk old *Id4-/-* mice [1]. The association of Pten expression with prostate cancer [15, 16], prompted us to investigate the expression of Pten at all developmental stages used in this study. Pten expression was absent/ below detection in *Id4-/-* at 25d, 3mo and 6mo (Fig. 4). Strong Pten reactivity was observed in wild at 3mo and 6mo wt (Fig. 4). Interestingly low Pten immune-reactivity was observed in 25d wt suggesting that robust Pten expression is a late event that may be associated with steeply rising androgen levels at puberty (25-30 days post-natal) resulting in prostate growth and terminal secretory differentiation (e.g. Pbsn secretion) that is complete by ~45 days post-natal. The loss of Pten expression is associated with increased pAkt expression that is not associated with increased Akt (data not shown) which is also consistent with our earlier reports on 6wk old mice [1]. These results may suggest that Pten expression is androgen regulated. However, direct evidence supporting the role of androgens in regulating Pten in the prostate epithelium is lacking.

### Luminal Differentiation of prostate epithelium

**Figure 5:**
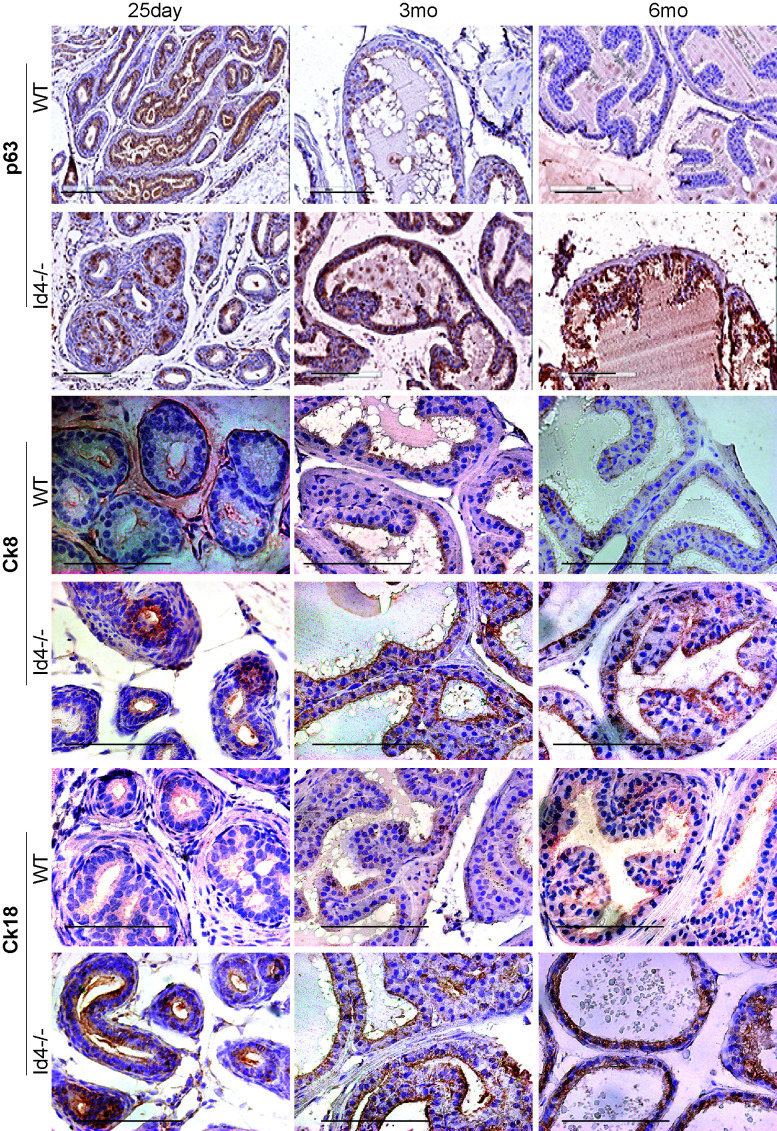
Immuno-histochemical localization of p63 (top panel), Keratin 8 (Ck8, middle panel), and Keratin 18 (Ck18, bottom panel) in *Id4-/-* (Id4 knockout) and wt (wild type) prostates from 25d, 3m and 6m old mice. The positive immune-reactivity is identifiable as brown staining. The bar in each panel is 100um. The blue staining represents nuclei stained with hematoxylin. Representative data from multiple fields and 3 different experiments is shown.

**Figure 6:**
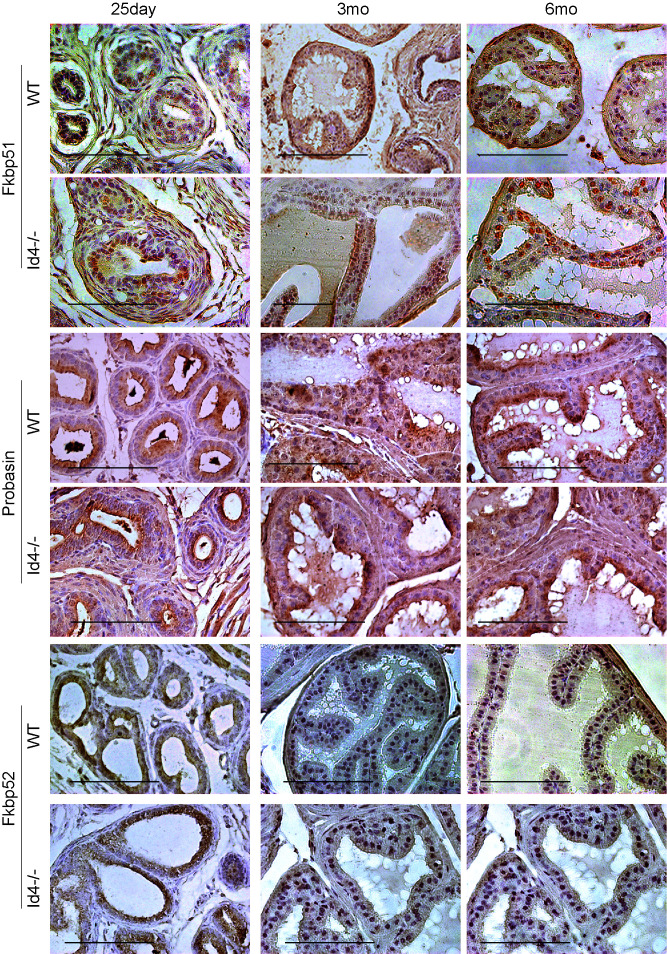
Immuno-histochemical localization of Fkbp51 (top panel), Probasin (middle panel) and Fkbp52 (bottom panel) in *Id4-/-* (Id4 knockout) and wt (wild type) prostates from 25d, 3m and 6m old mice. The positive immune-reactivity is identifiable as brown staining. The bar in each panel is 100um. The blue staining represents nuclei stained with hematoxylin. Representative data from multiple fields and 3 different experiments is shown.

The loss of Pten in *Id4-/-*, a major observation allowed us to compare the prostate phenotypes between *Id4-/-* and Pten null mice [17]. The Pten null mice is PSA/ Pbsn Cre driven hence the deletion is usually post-natal and around puberty which does not reflect the role of Pten in prostate development as observed in *Id4-/-* mice. Pten loss leads to increased stem/ progenitor cell populations without blocking differentiation [17]. However, the effect of Pten loss on cell lineage proliferation in prostate can be informative. It has been proposed that in normal prostate a basal epithelial cell compartment contains a small sub-population of cells that co-express the full spectrum of basal and luminal epithelial cell markers (Ck5, Ck14, Ck8, Ck18, Ck19, p63 and GSTpi) at all stages (embryonic to adult) [18]. These prostatic epithelial progenitor/ stem cells maintain a differentiation marker profile similar to that of the urogenital sinus epithelium. These progenitor/stem cells differentiate into mature luminal cells by maintaining Ck8 and Ck18, and losing all other makers [18]. The Ck18/ Ck8 expression between the wild type and *Id4-/-* was indistinguishable (Fig. 5). Interestingly, p63 positive cells were observed with increasing frequency in the *Id4-/-* mice as compared to the wild type. These results suggested that loss of *Id4-/-* leads to the expansion of a sub-population of cells that co-express a full spectrum of epithelial and basal markers. These, possibly progenitor/ stem cells are reminiscent of the expression profile in urogenital sinus. Thus, loss of Id4 may block the normal epithelial differential pathway. However, the presence of the secretory cytodifferentiation markers such as Pbsn (Fig. 6) suggest a unique cell fate pathway (secretory differentiation with basal and luminal markers) present in *Id4-/-* prostate cells.

### Ar is transcriptionally active in *Id4-/-* mice

Increase in nuclear Ar expression/ localization in *Id4-/-* mice (Fig. 4), which may represent increased transcriptional activity, led us to investigate the expression of Ar regulated genes in these mice. Fkbp51 [19] and Pbsn [20] are direct transcriptional targets of Ar. An increased expression of both Fkbp51 and Pbsn at all developmental stages studied was essentially similar in wild type and *Id4-/-* tissues suggesting that Ar is transcriptionally active and that the developmental defect is likely not due to altered Ar transcriptional activity (Fig. 6).

Ar transcriptional activity is also regulated by the chaperone Fkbp52 [21]. The Fkbp52-/- mice have several defects in reproductive tissues consistent with androgen insensitivity [22]. Among these defects are ambiguous external genitalia and dysgenic prostate. Similar expression levels of Fkbp52 between the wt and *Id4-/-* mice suggested that the prostate phenotype in *Id4-/-* mice was not directly due to altered Fkbp52 expression (Fig. 6). Moreover, androgen insensitivity was ruled out as seen by a robust expression of Fkbp51 and Pbsn in both wild type and *Id4-/-* mice (Fig. 6).

### Nkx3.1 expression in *Id4-/-* mice

**Figure 7:**
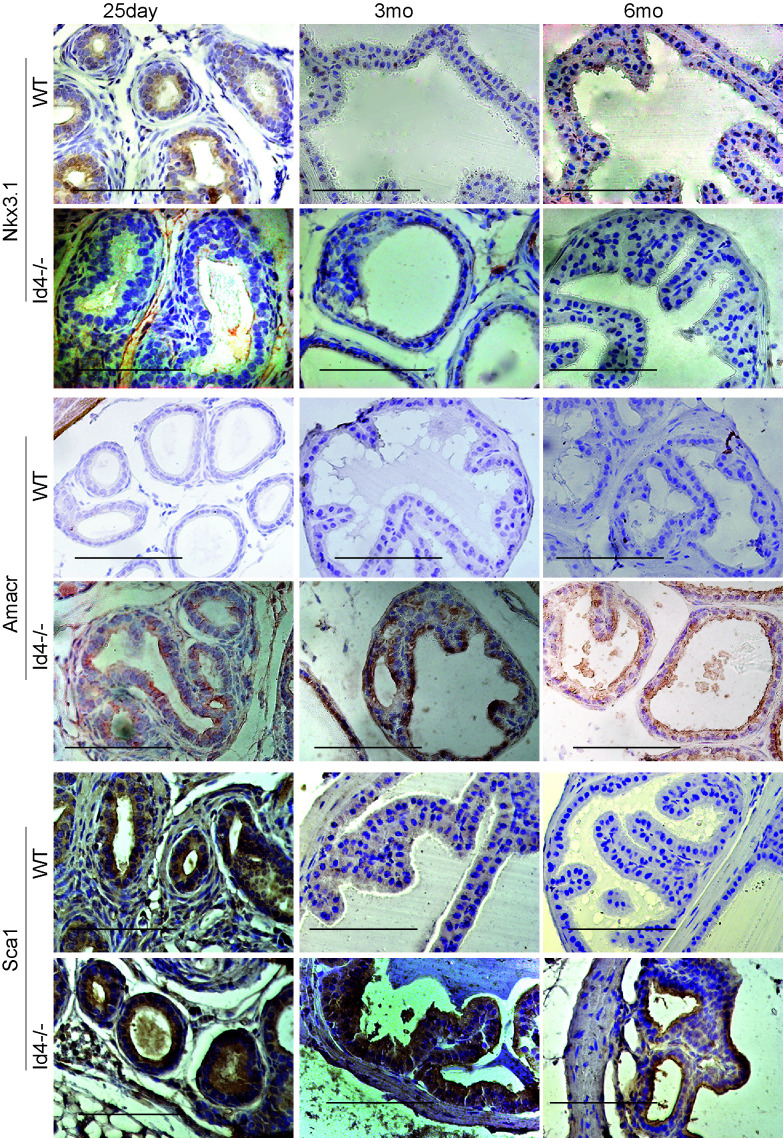
Immuno-histochemical localization of Nkx3.1 (top panel), Amacr (middle panel), and Sca1 (bottom panel) in *Id4-/-* (Id4 knockout) and wt (wild type) prostates from 25d, 3m and 6m old mice. The positive immune-reactivity is identifiable as brown staining. The bar in each panel is 100um. The blue staining represents nuclei stained with hematoxylin. Representative data from multiple fields and 3 different experiments is shown.

Nkx3.1 expression was essentially undetectable in *Id4-/-* mice at 6mo. Nkx3.1 positive cells (nuclei and cytoplasmic) were occasionally observed at 3mo *Id4-/-* prostate. Nkx3.1 expression was rarely observed at 25d prostate with the exception of some staining in inter-tubular tracts (most likely background staining) (Fig. 7). These results suggest that either Id4 regulates Nkx3.1 and that its expression precedes Nkx3.1 or Id4 loss blocks cellular differentiation pathway including expression of effectors that may be required for Nkx3.1 expression.

Nkx3.1 is an androgen regulated gene in the prostate [23, 24]. We had earlier shown that loss of Nkx3.1 in 6week old *Id4-/-* prostate was due the lack of Ar binding to the respective Androgen Response Element (ARE) sites on the Nkx3.1 promoter [1]. On the contrary, the expression of at least two other known Ar target genes Pbsn and Fkbp51 were expressed in *Id4-/-* mice, similar to the wt as discussed above (Fig. 4). These results suggest that nuclear Ar in *Id4-/-* mice might be active but appears to be selective in the context of its target genes. A similar AR activity is also observed in prostate cancer where AR fails to activate NKX3.1 [25] but promotes the expression of PSA [26] and FKBP51 [27] among others.

### AMACR expression as a marker of PIN lesions

AMACR is a highly specific prostate cancer/ PIN marker with no expression observed in normal epithelial cells/ Benign or hyperplastic cells [28]. AMACR expression was used as a definitive marker for identifying areas of PIN in *Id4-/-* mice. Surprisingly, AMACR expression was observed at higher levels in the *Id4-/-* mice as compared to almost no expression in the wild type mice (Fig. 7). AMACR expression was also observed in the regions that did not display hyperplastic/ dysplastic regions suggesting that these cells exhibit a pre-cancerous phenotype.

### Stem Cell population is present in *Id4-/-* mice prostates

Studies have demonstrated an increase in the percentage of stem cells (Sca1+)/ progenitor cells without a block in differentiation in the *PB-Cre4*; Pten^flox/flox^ model of high-grade PIN and prostate cancer [17, 29] mouse model. The loss of Pten in the *Id4-/-* model prompted us to investigate whether there was an enrichment of Sca1+ cells in *Id4-/-* mice which also lacks Pten, even though Pten loss is genetic and not Pbsn Cre driven in these mice. Interestingly, the Sca1+ reactivity was very high in *Id4-/-* mice prostates as compared to the wild type counterparts at all ages studied (Fig. 6). The Sca1 immunoreactivity was also observed in 25d old wild type prostates which decreased significantly by 3mo and rarely observed by 6mo (Fig. 7). The prostate epithelial cell differentiation markers such as Ck8/Ck18 (Fig. 5) and secretory markers such as Pbsn (Fig. 6) were expressed at similar levels as compared wild type mice at all ages between *Id4-/-* and wild type mice. These results suggested that loss of *Id4-/-* leads to the enrichment of cell population which co-express the full spectrum of luminal epithelial markers (Ck8 and Ck18) in addition to the expression of stem cell antigen Sca1 and basal marker p63.

### Id4 regulates Epithelial cell differentiation

**Figure 8:**
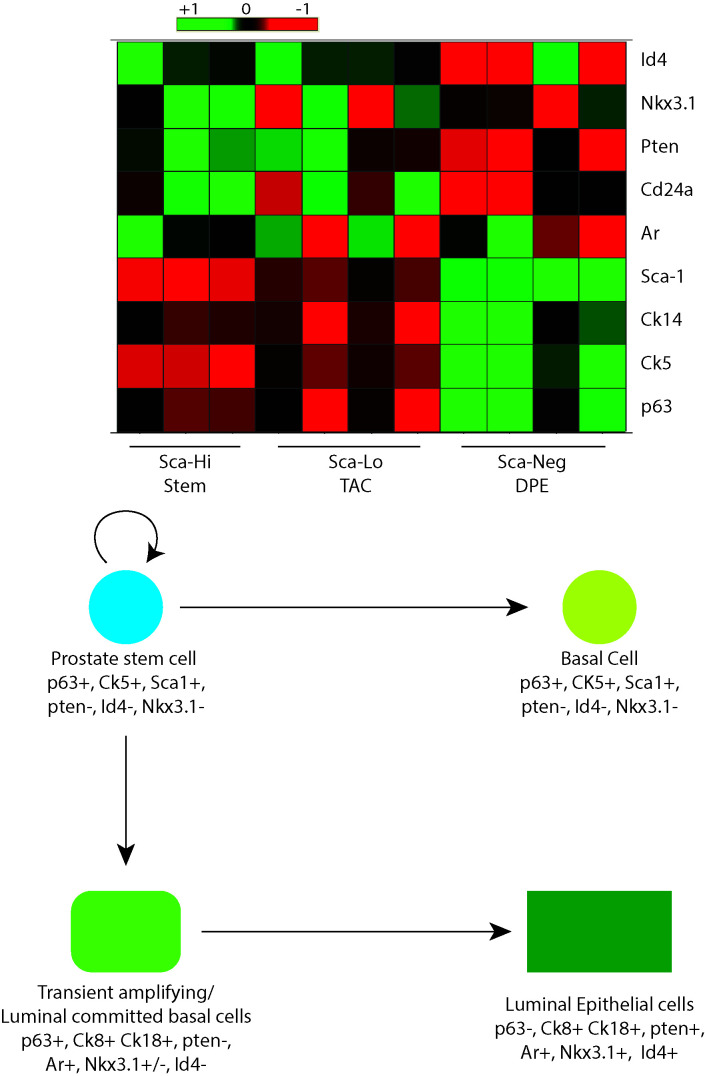
Top panel: Meta-analysis of selected genes in stem cell enriched population (Sca Hi) from 6-week-old normal prostate. The panel (indicated on the right) also demonstrates the expression of the respective genes in cell with low (Sca Low, Transient amplifying cells, TAC) and negative Sca expression 9Sca Negative, differentiated prostate epithelial cells, DPE). The scale bar on the top indicates level of expression. Bottom Panel: Schematic demonstrating the potential role of Id4 in regulating prostate epithelial cell differentiation and lineage. Consolidated data form the *Id4-/-* studies and panel shown above suggests that Id4 may be required for transient amplifying cells/ luminal committed basal cells to differentiate into luminal epithelial cells.

Two major observations made in this study: 1) continued expression of p63 and Sca1 along with 2) the expression of epithelial markers suggested that the cells in the *Id4-/-* prostate are likely intermediate epithelial cells or transient amplifying cells. In order to further explore the characteristics of this cell population, we analyzed the gene expression profile in Sca1 positive cells. This was accomplished be re-analyzing the previously published expression data of the selected genes in the Sca1 enriched population (prostatic stem cells) in prostatic ducts from the 6wk old mice [30] (GEO GSE15580). The heat map and hierarchical clustering analysis shown in Fig. 8, demonstrates that Id4 expression is only associated with Sca negative (Sca-Neg) cells which represent the differentiated epithelial cells. Interestingly, Pten is also expressed in Sca Neg but not in Sca1 enriched cells. On the contrary, Nkx3.1 and Ar expression is observed in Sca low transient amplifying/ intermediate luminal epithelial cells. The transient amplifying cells also express the basal cell markers *p63* and *Ck5*. These results independently confirm our results and strongly suggest that loss of Id4 arrests the cells at the transient amplifying/ intermediate luminal epithelial stage.

### Id4 expression in *Nkx3.1-/- *mice prostates

**Figure 9:**
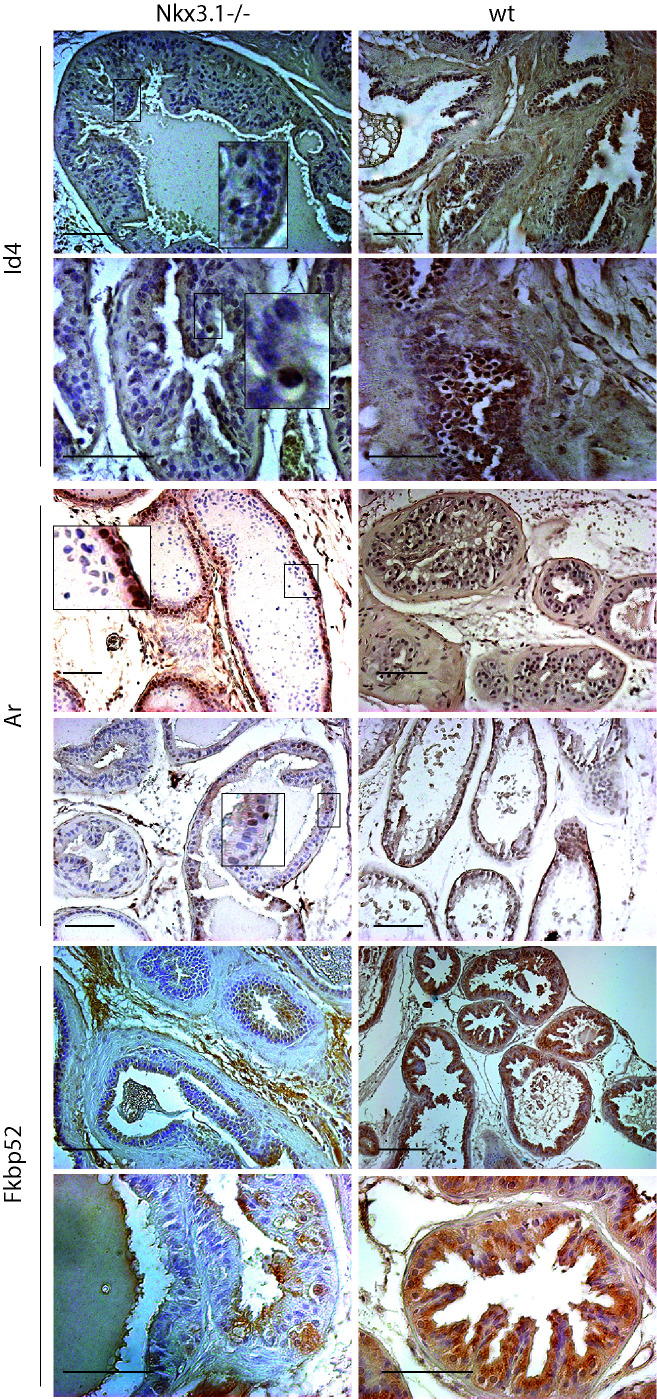
The expression of Id4, Ar and Fkbp52 in adult Nkx3.1 -/- (knockout) and wild type mice. The insets represent an enlarged boxed region. The positive immune-reactivity is identifiable as brown staining. The bar in each panel is 100um. The blue staining represents nuclei stained with hematoxylin. Representative data from multiple fields and 3 different experiments is shown.

To further explore and establish the Id4-Nkx3.1 nexus, we investigated the expression of Id4 in Nkx3.1 mouse prostates. As shown in Fig. 9, Id4 expression was low to negligible in *Nkx3.1-/- *mice prostate as compared to their wild type counterparts. Even when Id4 was present in epithelial cells, the expression was dramatically lower than the Id4 expression observed in wild type prostate epithelial cells (Fig. 9). Interestingly a peculiar Ar expression profile was observed in the *Nkx3.1-/- *prostate as compared to wt and *Id4-/-*prostate. As compared to the near uniform (primarily nuclear) expression of Ar observed in wt, the Ar expression in *Nkx3.1-/- *was lobe specific. In the ventral lobe, Ar expression was present in all cells close to the basement membrane but absent in the cells towards the lumen (Fig. 9). In other lobes (dorsal, shown) Ar expression was present only in a small subset of cells. Interestingly, the expression of Ar target gene Pbsn is significantly lower in *Nkx3.1-/- *mice as compared to wild type [31]. In contrast, Pbsn was expressed in the *Id4-/-* mice (Fig. 6). The prostate development between *Nkx3.1-/-* and *Id4-/-* mice therefore appears distinct. In adult Nkx3.1 knockout mice, prostatic lobes demonstrate a significant reduction (up to 60%–75% of wild type) in ductal tip number that is evident as early as 10–11 days of age, when ductal branching is nearly complete, but pubertal growth has not yet begun in the wt [12]. In contrast, the overall sizes and wet weights of the prostatic lobes in the *Nkx3.1-/-* are similar to wild type. Reduced ductal branching without an accompanying decrease in overall size, suggests reduced ductal complexity in Nkx3.1 mutant prostates [11]. On the contrary, the wet weight of prostates in *Id4-/-* are significantly lower in part due to reduced ductal branching. Thus, the prostate phenotype in *Id4-/-* mice is not only due to loss of Nkx3.1 expression (ductal branching) but due to loss of other factors (e.g. Pten) that are involved in prostate development.

Fkbp52 expression was also determined in *Nkx3.1-/- *mice in order to explore whether Ar activity is altered, even though the dysgenic prostate were not observed as in *Fkbp52 -/-* mice [22]. As shown in Fig. 7, Fkbp52 expression was reduced in *Nkx3.1-/- *mice as compared to the wild type prostate. Though a general loss of expression was not apparent, but as opposed to uniform strong expression observed in the wild type, the Fkbp52 expression in *Nkx3.1-/- *was present in only localized regions within the same tubule. The results suggested that Fkbp52 expression is attenuated in *Nkx3.1-/- *mice which may result in altered Ar activity that could be reflected in lower Pbsn expression in *Nkx3.1-/- *mice [31].

## Discussion

The HLH transcriptional regulator ID4 is a relatively new player in the prostate development and prostate cancer landscape. Experimental and meta-analysis has consistently directed towards the putative role of ID4 as tumor suppressor in prostate cancer and more importantly as a key regulator of mammalian prostate development as reported in this and our earlier studies [1, 5, 6, 9, 32].

Prostate development is a complex process that involves coordination of multiple signaling pathways including endocrine, autocrine and transcription factors. The prostate gland arises from the urogenital sinus (UGS) which itself appears around 13 days post coitus (dpc). Prostate development from the UGS is initiated at about 17.5dpc characterized by the growth of prostatic epithelial buds from the urogenital epithelium (UGE) into the urogenital mesenchyme (UGM) and is initially dependent on the circulating androgens produced by fetal testis (13-14dpc). Up until birth, the UGS/ rudimentary prostate outgrows as solid epithelial buds in a precise spatial pattern but without any branching. In the neonatal prostate, the buds elongate, bifurcate and send out branches. The epithelial, mesenchymal and stromal cytodifferentiation occurs during the first 2-3 weeks after birth as seen by the expression of cell type specific expression markers (epithelial/ luminal: Ck8, Ck18, basal: Ck5, Ck14 and p63). The branching morphogenesis is almost entirely complete by 2 weeks after birth. During this time testosterone is low and there is only a modest increase in prostate weight. Androgen levels steeply rise at puberty (25-30 days post-natal) resulting in prostate growth and terminal secretory differentiation (e.g., Pbsn secretion) that is complete by ~45 days post-natal. The prostate development can thus be divided into 3 broad stages: Induction (embryonic), branching morphogenesis (pre-pubertal) and secretory differentiation (pubertal/ post-pubertal) [12, 33, 34]. From the data shown in this study, it is evident that loss of Id4 has no effect on the induction of prostate gland formation. However, branching morphogenesis in *Id4-/-* mice is clearly reduced, whereas the secretory differentiation largely seems to be intact based on standard secretory marker profile. The *Id4-/-* model clearly represents a complex prostate phenotype with stem cell markers being co-expressed with epithelial differentiation and secretory markers in an under-developed prostate.

There are two major categories of morpho-regulatory genes involved in prostate development, the nuclear transcription factors that include common and organ specific homeobox genes (including Nkx3.1) and secreted signaling ligands encoded by a small number of conserved multigene families. The ligand activated nuclear transcription factor, androgen receptor clearly has a primary role in prostate development. The androgen receptor continues to be expressed during the earliest stages of prostate development from UGS at 13 dpc through adult life. Loss of Ar by any means results in the complete loss of the prostate [34]. That the prostate was present and demonstrated some degree of branching morphogenesis and cyto-differentiation (Pbsn, Fkbp51) clearly suggests a functional Ar in Id4/- mice.

Nkx3.1, a homeobox gene also plays an essential role in prostate development. Nkx3.1 expression is first detected in the urogenital sinus epithelium at 15.5 dpc, which is at least 2 days before the emergence of prostate buds/ prostate formation suggesting that regions of the urogenital sinus epithelium may have a differential capacity to form prostate [11]. These emerging prostatic buds are marked by expression of Nkx3.1 in the prostatic epithelium [11]. During murine embryogenesis, androgen receptors are located in the UGM, whereas postnatally they are found in both the mesenchyme and epithelium. Although the initial appearance of Nkx3.1 expression in the prostatic epithelium precedes that of the androgen receptor, the subsequent expression of Nkx3.1 is dependent on androgen signaling, as shown in tissue recombination assays [11]. Nkx3.1 is essential for normal morphogenesis and function of the prostate, whereas its inactivation leads to decreased branching with smaller ductal tips, prostatic epithelial hyperplasia and dysplasia in part due to increased proliferation that model a preneoplastic condition. Although Nkx3.1 is the earliest known differentiation marker of the prostate epithelium, it must cooperate with other regulatory genes, as its loss of function does not result in complete failure of prostate formation. Many of the features of the *Id4-/-* prostate are also similar to *Nkx3.1-/- * phenotype such as decreased ductal branching and increased proliferative capacity, however, there are many differences also. The gross hyperplasia and dysplasia are largely absent and are visible marginally only at 3-6months in dorsal prostate lobes. The defect of *Id4-/-* seems largely developmental since primitive UGS with canalization but no ductal branching is present at 25days that persists into 3 and 6 months. Thus, Id4 may represent a critical factor that regulates prostate morphogenesis. Interestingly, Id4 expression is not completely abolished in the *Nkx3.1-/-* mice suggesting that low Id4 expression in the *Nkx3.1-/- *may be necessary for maintaining the branching morphogenesis. Although we did not perform Id4 expression in the embryonic prostate however, meta-analysis on dpc 17 prostate indicated high Id4 expression levels suggesting an early event ([35] GDS5233 NCBI Geo Dataset), similar to Nkx3.1 expression in prostate morphogenesis. It is likely that Id4 may be expressed earlier than Nkx3.1 in the UGS implying that Id4 may be required for Nkx3.1 expression. The meta-analysis on Sca1 enriched cell population suggested otherwise, i.e. Nkx3.1 expression may be earlier at least in a subset of transient amplifying Sca1 low cells. ID4 is not a transcription factor per se but modulates the activity of other transcription factors such as bHLH proteins [36], hence most of its regulatory function appears to be indirect through interaction with other regulatory proteins e.g. with bHLH proteins, chaperons such as Fkbp52 [7] and other transcription factors such as p53 [8]. We have earlier shown that decreased Nkx3.1 expression in *Id4-/-* is in part in due to decreased binding of the androgen receptor to the ARE site on the Nkx3.1 promoter, suggesting that the presence of Ar may not always translate into Ar transcriptional activity [1]. For example, Ar could activate Fkbp51 and Pbsn but not Nkx3.1 suggesting a shift/ specificity towards certain ARE elements in the promoters. Contrary to its tumor suppressor role, some studies demonstrated that NKX3.1 may act as a pro-survival factor in prostate in collaboration with AR. The enrichment of NKX3.1 motifs at androgen receptor binding sites (ARBS) suggested that NKX3.1 is a collaborative factor of AR and a pro-survival factor [37]. Furthermore, AR can act as a transcriptional repressor on a global scale. The pioneering factors and ARE motifs located near the AR-induced genes are also involved in recruiting AR to repressed genes. Once bound to target loci, the repressive function of AR, however, is dictated, at least in part, by the Polycomb group protein enhancer of zeste 2 polycomb repressive complex 2 (EZH2) and consequent H3K27 trimethylation [38]. Thus, discovering genome wide ARE sites occupied by Ar in the *Id4-/-* may be required to fully understand how loss of Id4 modulates Ar transcriptional activity. These ARE sites could be adjacent to other regulatory region, exhibit locus specific chromatin remodeling or selective loss/ gain of chaperons/ modifications that may alter the specificity of the Ar.

Low expression of Id4 in Nkx3.1 knockout may also be linked to NKX3.1 being collaborative factor for AR activity. Since Id4 is also AR regulated (although no known ARE sites are present in the proximal ID4 promoter) [39], the loss of NKX3.1 may modulate the activity of transcription factors that may ultimately regulate AR. Alternatively, loss of ID4 may epigenetically silence NKX3.1 promoter. Our data suggests that NKX3.1 and ID4 may exist in a feed forward loop at least during prostate morphogenesis. Alternatively, decreased in Nkx3.1 may simply be due to a developmental arrest in the *Id4-/-* prostate.

Similar to Nkx3.1, the expression of Pten was also below detection levels in Id4 KO mice. Pten is a major tumor suppressor that is frequently mutated/ deleted in many human cancers including prostate. In prostate cancers, PTEN deletions/ mutations are found in 20% primary prostate cancers and 50% of castration resistant prostate cancer [40, 41]. In mice, Pten-/- deletion is early embryonic lethal. The Pten heterozygous +/- develop PIN lesions with near 100% penetrance with a rather long latency of approximately 10 months. Of note is that even after such a long latency period these PIN lesions never progress to metastatic disease [42], suggesting that LOH of PTEN alone is not sufficient for prostate tumor progression and metastasis. Elegant studies by Trotman et al., demonstrated that further reduction of Pten in a Pten hypomorphic allele (hyp/-) mouse model accelerates tumor progression dramatically, eventually resulting in high-grade PIN and locally invasive carcinoma [15]. The low penetrance of the invasive prostate cancer in these mutants strongly suggested that additional events and/or that other genetic changes are necessary to develop prostate cancer.

Sca1 reactivity in *Id4-/-* prostate clearly suggests that Id4 expression is ultimately required for the complete luminal cyto-differentiation. Sustained expression of basal cell marker p63 and epithelial differentiation markers Ck8/ 18 and Pbsn suggest a unique developmental defect wherein a full spectrum of marker from stem cell to epithelial are co-expressed in the *Id4-/-* prostatic epithelial cells. This marker profile may be similar to the cell differentiation marker profile of the urogenital sinus epithelium. Id4 can promote the differentiation of these progenitor stem cells into mature luminal cells by maintaining CK8 and CK18 [17, 18]. The meta-analysis of Sca1 population from adult prostatic ducts clearly supports this observation. Thus loss of Id4 leads to the arrest of the transient amplifying cells or more aptly of the luminal committed basal cells that are characterized by Nkx3.1+/-, Pten-, Ar+, p63+, Ck8+, Ck5+ marker profile (Fig. 8 and [43]). Disruption Id4 promotes a surplus of luminal committed basal cells that could not differentiate due to lack of Id4 induction.

The *Id4-/-* mice represented a complex phenotype in which at least two major tumor suppressors Nkx3.1 and Pten were below detection by conventional immune-histochemistry. In spite of this phenotype, widespread PIN lesions with a significant degree of penetrance were not noted nor any evidence of tumors. PIN lesions may be described as proliferation and stratification of epithelial cells with atypical nuclei and with progressive neoplastic epithelial growth, the stratification region can acquire a tufting, micropapillary, or cribriform growth pattern. In fact, epithelial stratification and a tufting pattern was readily visible in the dorsal prostate lobe of the 6mo old *Id4-/-* mice. These results suggested that loss of *Id4-/-* may results in low penetrance PIN with a latency of about 6 months. Our study suggests that PIN lesions are not only recognized by atypical proliferation and tufting, the visible hallmarks of PIN, but can also be recognized as pre-neoplastic cells as seen by Amacr reactivity as early as 25d. Whether these tubules/ cell will eventually develop show visible PIN lesions during later life (i.e. beyond six months) remains to be seen. Nevertheless, our results strongly support the notion that a sub-population of cells that may eventually develop PIN can be recognized very early based on Amacr reactivity. These sub-populations of cells may also demonstrate sca-1 expression, a marker for mouse stem cells. Meta-analysis suggested that the fraction Sca-1 is high in Id4 non-expressing cells. Progression of these high Sca-1 cells (stem cells) to low Sca-1 (transient) to Sca1 negative (differentiated) is associated with progressive increase in Id4 expression.

In conclusion the *Id4-/-* knockout presents a complex prostate phenotype. Loss of Id4 results in altered prostate development but also leads to or promotes some PIN like lesions with maintaining its stem cells, that are supported both by morphological and specific marker studies. Following potential *Id4-/-* dependent mechanisms can be conceptualized. First, the altered androgen-receptor – Id4 interaction pathway in which Id4 is required to promote androgen dependent differentiation program. This mechanism is supported by the Id4 dependent Nkx3.1 expression as shown in normal prostate epithelial cells. Second, a stem cell hypothesis where in Id4 is required to maintain or influence the timing of differentiation of a specific stem cell population. Alteration in any of these pathways could result in abnormal prostate and reproductive tract development and may establish gene expression signatures that favor (PTEN, NKX3.1, Id1, Myc) or restrain (Akt) development of prostate gland and pre-cancerous lesions.

## Materials and Methods

### Animals

The *Id4-/-* mice were generated by targeted replacement of the endogenous Id4 locus with the green fluorescent protein (GFP) coding sequence as described elsewhere [4]. These mice were housed in Geisel Medical School at Dartmouth. All animal studies were approved by the Institutional Animal Care and Use Committee, Geisel Medical School at Dartmouth. The mice were sedated using a lethal dose of tribromoethanol (TBE) followed by terminal perfusion with 10% acetate buffered formalin. The prostates from 25 days, 3 months and 6 months old *Id4-/-* and Id4+/+ mice fixed in buffered formalin were provided by Dr. Matthew Havrda (Geisel School of Medicine, Hanover, NH, USA). The fixed tissues were paraffin embedded and used for all histological studies as described below.

Five micron sections of Formalin fixed paraffin embedded Nkx3.1-/- prostate were kindly provided by Dr. Sarki A Abdulkadir, Professor of Urology, Feinberg School of Medicine, Northwestern University.

### Histological/ Immuno-histological analysis

All histological and immuno-histochemical analyses were performed on 5um sections. The sections were stained with hematoxylin and eosin using standard procedures.

Immuno-histochemistry was performed following standard procedures as described earlier [1]. Briefly, antigens were retrieved in an autoclave (in 0.01 M sodium citrate buffer pH 6.0) at 121C/20 psi for 30 min). Following antigen retrieval, the endogenous peroxidase activity and nonspecific binding sites were blocked in 3% H2O2 and 10% Goat serum respectively. The blocked sections were then incubated overnight at 4°C with either of the following antibodies: Androgen receptor (Cell Signaling, cat#153P), Pten (Cell Signaling, cat#9559), NKX3.1 (Thermo Scientific, cat#16906), Sox9 (Novus biological, NB-100-2202), Id4 (BioCheck, BCH-9/82-12), AMACR (AMACR Novus cat # NBP 1-28884), CK8 (Proteintech Cat # 10384-1-AP), CK-18 (Proteintech Cat # 18708-1-AP), FKBP4 (Proteintech Cat# 10655-1-AP). All the antibodies were mono-reactive, that is a single reactive band was observed in western blot using total cell lysate from prostate cancer cell lines LNCaP, DU1545 and PC3. Non-specific binding of the secondary antibodies was evaluated using respective normal IgGs (data not shown).

The sections were then incubated with secondary antibody (goat anti-rabbit (#32260) or goat anti-mouse (#32230) -HRP, Thermo Scientific) for 1 hour. The slides were stained with DAB for 2 min, counterstained with hematoxylin and mounted with Immuno-mount (Thermo Scientific). The slides were either examined and photo-micrographs taken using the Zeiss microscope with an AxioVision version 4.8 imaging system or digitized on the Leica Aperio and viewed on the Aperio ImageScope. The H&E sections from knockout, and wild type mice were examined by Drs. Saini and Jain (pathologist and oncologist respectively).

### Microarray Gene Expression

Affymetrix CEL files from the published dataset GSE15580 were downloaded from the National Center for Biotechnology Information (NCBI) Gene Expression Omnibus database (https://www.ncbi.nlm.nih.gov/geo/download/?acc=GSE15580&format=file). The files were processed with Affymetrix Expression Console (EC) Software Version 5.0 using the default MAS5 3' expression workflow. The raw data was Z-Score normalized with Spotfire DecisionSite software. For the probe sets presented in Fig. 8, hierarchical clustering was performed and resulted in the 3 groups depicted below the heatmap.
